# Case Report of an Infrequent Complication of Infective Endocarditis: The Complete Detachment from the Aorta of the Previous Vein Graft Anastomosis

**DOI:** 10.1055/s-0039-1678554

**Published:** 2019-04-24

**Authors:** Laura Varela Barca, Jose López Menéndez, Ana Redondo Palacios, Jorge Rodríguez-Roda Stuart

**Affiliations:** 1 Department of Cardiovascular Surgery, Ramon y Cajal Hospital, Madrid, Spain

**Keywords:** infectious endocarditis, composite aortic graft, combined surgery, mediastinal collection

## Abstract

The authors report an unusual complication of acute infective endocarditis found in a 70-year-old man with a previous history of two cardiac surgery procedures. During median sternotomy, a massive bleeding occurred. The bleeding was contained within the mediastinum and originated from the 10-year-old anastomosis of the saphenous vein to a composite graft, which was completely detached due to infective endocarditis of the aortic graft.

## Introduction

Infective endocarditis (IE) is an important cause of cardiovascular pathology that, despite diagnostic and therapeutic advances, continues to be a serious disease that leads to a high morbidity and mortality. 1 The presence of local complications (such as periannular tissue destruction or abscess) increases the complexity and mortality associated with IE surgery. 2 An episode of IE affecting a composite graft after aortic root replacement can be a life-threatening complication. 3 4 Moreover, redo procedures after a previous coronary artery bypass grafting (CABG) surgery, with a patent internal mammary artery (IMA), may significantly increase the risk of the surgical procedure.

We report the case of a 70-year-old man with an episode of IE affecting a composite aortic root and mitral prosthesis, who had an extremely high surgical risk, in whom a previous saphenous vein graft was found to be detached from the aortic graft because of endocarditic involvement, forming a huge collection of blood in the anterior mediastinum.

## Case Presentation

A 70-year-old man was emergently referred to the Cardiac Surgery Department due to a confirmed diagnosis of acute IE. The patient had a previous clinical history of arterial hypertension and atrial fibrillation. He had two previous cardiac surgery procedures. He underwent, 10 years ago, a full aortic root replacement with a composite graft (Dacron graft with mechanical valve, Bentall-De Bono procedure) associated with triple coronary bypass (IMA to anterior descending artery and vein grafts to intermediate branch and posterior descending artery).

The second procedure was performed 6 months before the current episode, when the patient suffered from a native mitral valve IE episode ( *Staphylococcus epidermidis* ). He was admitted in cardiogenic shock with confirmed endocarditic involvement of the mitral valve. The critical preoperative status did not permit a preoperative angiogram. No signs of myocardial ischemia were present, so the patient underwent an emergent mitral valve replacement by a mechanical prosthesis performed through a right thoracotomy approach, to avoid possible complications related to the previous coronary grafts. The postoperative course was uneventful, and the patient was discharged home after completing 6 weeks of intravenous antibiotic treatment with daptomycin and rifampicin.

The current episode started when the patient was readmitted to our institution in a critical clinical state, with congestive heart failure and sepsis. The patient presented with persistent fever, dyspnea, orthopnea, and paroxysmal nocturnal dyspnea. On physical examination, no peripheral stigmata of endocarditis were found. A diastolic murmur was heard along the left sternal border. Three blood cultures were positive for *Staphylococcus aureus* . Antibiotic treatment was initiated with intravenous oxacillin, rifampicin, and gentamicin. Complete imaging studies were conducted.

**Fig. 1:**
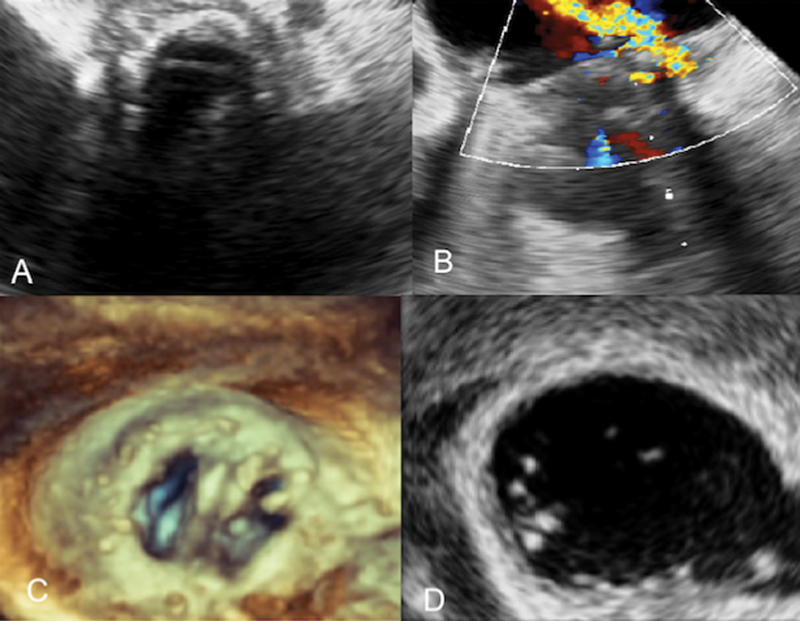
Transesophageal echocardiography. ( **A** ) Prosthetic aortic valve with a perivalvular abscess. ( **B** ) Mitral periprosthetic leak. ( **C** ) Mitral valve 3D reconstruction. ( **D** ) Vegetations on the mitral prosthetic valve.

Transesophageal echocardiography revealed a mitral peri-prosthetic leak that caused severe mitral valve regurgitation. There were vegetations on both the aortic and mitral prosthetic valves. Annular involvement was diagnosed, with the presence of a large perivalvular aortic abscess ( Fig. 1 ).

The coronary angiography showed atherosclerotic coronary disease of the anterior descending and distal circumflex arteries. All the previous bypasses were not patent. It was not possible to selectively make an injection in the previous vein grafts. Comparison with the previous angiogram was impossible, as no coronary angiography was performed for the previous mitral IE surgery due to the urgency of the intervention.

**Fig. 2:**
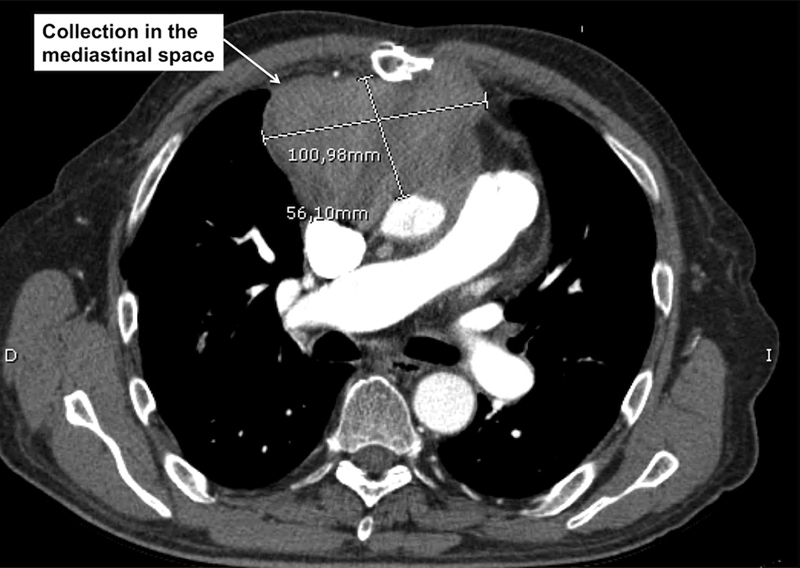
Computed tomography scan with intravenous contrast. Axial section showing a big collection in the mediastinal space.

A preoperative thoracic computed tomographic (CT) scan was performed, which revealed a huge collection of unknown origin in the anterior mediastinal space (100 × 55 × 75 mm), adjacent to the Dacron composite graft, in its anterior aspect, and in close contact with the thoracic wall ( Fig. 2 ). This was a clearly delimited collection, with dense and heterogeneous content. As there were no images of flow of contrast inside this collection, it was suspected to be purulent material. Soft tissue attenuation was described around the mechanical aortic valve, suggestive of active IE. After a period of stabilization (13 days) and antibiotic therapy, the patient underwent surgery.

Before opening the sternum, because of the suspected high risk of rupture, arterial cannulation was performed in the axillary artery and venous cannulation in the femoral vein. Cardiopulmonary bypass and hypothermia were established. During the sternal opening, a massive acute bleeding occurred. The bleeding was partially controlled by occlusion of the bleeding point by digital pressure through the partially opened sternum. After completing the sternal opening, the site of bleeding was identified. It was caused by the complete detachment from the aorta of the previous vein graft anastomosis, due to endocarditic involvement. The institution of cardiopulmonary bypass before sternal opening allowed the medical team to maintain a stable hemodynamic situation, and the bleeding site was controlled.

All the affected tissues were excised, including debridement of all infected and necrotic regions. The patient underwent a mitral valve replacement and a full root replacement with a mechanical composite graft (Medtronic Inc.). It was not possible to mobilize the native coronary ostia because of the firm adhesions due to the previous surgeries and the severe IE, so the new Bentall-De Bono procedure was performed with the Cabrol modification, 5 with an 8-mm Dacron graft. As there was no critical ischemic heart disease in the preoperative checkup, no new coronary bypasses were constructed. Despite the prolonged aortic cross clamp time (206 minutes) and pump time (300 minutes), no temporary circulatory support was necessary. The patient was transferred to the intensive care unit in a stable hemodynamic situation, with low-dose inotropic drugs and good perfusion.

During the first 24 hours after the intervention, it was possible to reduce the dose of inotropic support because of the stable hemodynamic state of the patient. Unfortunately, on the second postoperative day, the patient had fever and worsening of infectious parameters. The patient suffered from an episode of septic shock due to *Klebsiella pneumoniae* (Carbapenemases producer) that triggered multiorgan failure with acute renal failure, coagulopathy due to low flow hepatic failure, intestinal ischemia, and respiratory involvement. He died due to an acute multifactorial etiology shock refractory to medical treatment.

## Discussion

The presence of IE perivalvular complications increases the complexity associated with IE surgery. 2 Periannular tissue destruction, abscess formation, prosthesis dehiscence, presence of vegetation, and pseudoaneurysm formation are serious complications associated with a high mortality rate. 4 In addition, composite graft IE after aortic root replacement can be a life-threating complication, 3 and recurrent valve IE remains a surgical challenge because a more radical and aggressive surgical treatment is required to prevent a recurrent infection.

We report a case with an extremely high surgical risk, which was a second episode of IE, with an estimated 30-day mortality by EuroSCORE I and II of 81.6 and 42.6%, respectively. The case was discussed in the multidisciplinary endocarditis team, with the collaboration of the cardiologist, microbiologist, and cardiac surgeon, and despite the extreme surgical risk, the decision to proceed with surgery was accepted, as it was the only chance for survival. Surgery appeared to be the only possible strategy based on the clinical management guides. 1 6

Moreover, we found a very infrequent complication of IE: the total detachment of a previous vein graft anastomosis ought to an endocarditic involvement of the ascending aortic graft, with the formation of a large collection of blood contained by the adhesions of the previous surgeries. Prosthesis detachment due to IE affection 7 and ischemia due to abscess compromise 8 have been described, but we found no literature describing bypass graft involvement by infection.

Owing to the absence of similar reports in the previously published literature, we consider that this case is a good example of an extreme IE affectation. Moreover, our findings may help the planning of the surgical strategy in the unusual case of a mediastinal collection in an IE patient affecting a previous full-root graft and saphenous vein grafts. Cannulation for cardiopulmonary bypass before opening the sternum allowed the surgical team to control the potentially fatal complication of massive bleeding. Peripheral cannulation prior to sternal opening could be considered mandatory in advanced IE cases, when the destruction of outer heart structures is suspected.

## References

[1] Habib G Lancellotti P Antunes M J 2015 ESC guidelines for the management of infective endocarditis Eur Heart J 2015 36 44 3075 3128 26320109 10.1093/eurheartj/ehv319

[2] Lee S Chang B-C Park H K Surgical experience with infective endocarditis and aortic root abscess Yonsei Med J 2014 55 05 1253 1259 25048482 10.3349/ymj.2014.55.5.1253 PMC4108809

[3] Ramos A García-Montero C López-Menéndez J Endocarditis in patients with ascending aortic prosthetic graft: a case series from a national multicentre registry Eur J Cardiothorac Surg 2016 50 06 1149 1157 27283156 10.1093/ejcts/ezw190

[4] Apaydin A Z Posacioglu H Islamoglu F Degirmenciler K Durmaz I Composite graft endocarditis: repair with a mechanical prosthesis Tex Heart Inst J 2004 31 03 306 308 15562854 PMC521777

[5] Cabrol C Gandjbakhch I Cham B [Aneurysms of the ascending aorta; total replacement with reimplantation of the coronary arteries (author's transl)] [in French] Nouv Presse Med 1978 7 05 363 365 345234

[6] Prendergast B D Tornos P Surgery for infective endocarditis: who and when? Circulation 2010 121 09 1141 1152 20212293 10.1161/CIRCULATIONAHA.108.773598

[7] Yamazato A Aoshima M Nishimura N Ban T [A case report of emergency Bentall re-operation] [in Japanese] Kyobu Geka 1989 42 09 778 781 2615124

[8] Hussein N Qamar S Abid Q Systemic aspergilloma post aortic root surgery following coronary artery stenting: diagnostic and management dilemma BMJ Case Rep 2015 2015 bcr2014207702 10.1136/bcr-2014-207702 PMC4458591 26025972

